# Systematic Selection of Reference Genes for the Normalization of Circulating RNA Transcripts in Pregnant Women Based on RNA-Seq Data

**DOI:** 10.3390/ijms18081709

**Published:** 2017-08-04

**Authors:** Stephen S. C. Chim, Karen K. W. Wong, Claire Y. L. Chung, Stephanie K. W. Lam, Jamie S. L. Kwok, Chit-Ying Lai, Yvonne K. Y. Cheng, Annie S. Y. Hui, Meng Meng, Oi-Ka Chan, Stephen K. W. Tsui, Keun-Young Lee, Ting-Fung Chan, Tak-Yeung Leung

**Affiliations:** 1 Department of Obstetrics & Gynaecology, Faculty of Medicine, The Chinese University of Hong Kong, Shatin, New Territories, Hong Kong, China; wkwkaren@cuhk.edu.hk (K.K.W.W.); steplamkw@gmail.com (S.K.W.L.); cylai@cuhk.edu.hk (C.-Y.L.); yvonnecheng@cuhk.edu.hk (Y.K.Y.C.); anniehui@cuhk.edu.hk (A.S.Y.H.); mengmengmumu@gmail.com (M.M.); cokchan@cuhk.edu.hk (O.-K.C.); 2 School of Life Sciences, Faculty of Science, The Chinese University of Hong Kong, Shatin, New Territories, Hong Kong, China; clairechung@link.cuhk.edu.hk (C.Y.L.C.); tf.chan@cuhk.edu.hk (T.-F.C.); 3 Hong Kong Bioinformatics Center, The Chinese University of Hong Kong, Shatin, New Territories, Hong Kong, China; jamie_slk@link.cuhk.edu.hk (J.S.L.K.); kwtsui@cuhk.edu.hk (S.K.W.T.); 4 School of Biomedical Sciences, Faculty of Medicine, The Chinese University of Hong Kong, Shatin, New Territories, Hong Kong, China; 5 Department of Obstetrics & Gynaecology, Division of Maternal and Fetal Medicine, Kangnam Sacred Heart Hospital, College of Medicine, Hallym University, Seoul 07441, Korea; mfmlee@hallym.ac.kr

**Keywords:** reference target, reverse-transcriptase polymerase chain reaction (RT-PCR), normalization, transcriptomics, blood biomarkers, geNorm, NormFinder, Removal of Unwanted Variation from RNA-seq data (RUVSeq), technical variation, denoise

## Abstract

RNA transcripts circulating in peripheral blood represent an important source of non-invasive biomarkers. To accurately quantify the levels of circulating transcripts, one needs to normalize the data with internal control reference genes, which are detected at relatively constant levels across blood samples. A few reference gene candidates have to be selected from transcriptome data before the validation of their stable expression by reverse-transcription quantitative polymerase chain reaction. However, there is a lack of transcriptome, let alone whole-transcriptome, data from maternal blood. To overcome this shortfall, we performed RNA-sequencing on blood samples from women presenting with preterm labor. The *coefficient of variation* (*CV*) of expression levels was calculated. Of 11,215 exons detected in the maternal blood whole-transcriptome, a panel of 395 genes, including *PPP1R15B*, *EXOC8*, *ACTB*, and *TPT1*, were identified to comprise exons with considerably less variable expression level (*CV*, 7.75–17.7%) than any *GAPDH* exon (minimum *CV*, 27.3%). Upon validation, the selected genes from this panel remained more stably expressed than *GAPDH* in maternal blood. This panel is over-represented with genes involved with the actin cytoskeleton, macromolecular complex, and integrin signaling. This groundwork provides a starting point for systematically selecting reference gene candidates for normalizing the levels of circulating RNA transcripts in maternal blood.

## 1. Introduction

Quantitative polymerase chain reaction (qPCR) is a standard method for the quantification of nucleic acid sequences [1,2]. Combined with a prior step of reverse transcription (RT), RT-qPCR has become a well-established technique to quantify the level of any mRNA transcript in a sample [3]. To control for experimental error between samples that can be introduced at a number of stages throughout the procedure, the normalization of the RT-qPCR data is essential before analysis. This is usually achieved by the use of a reference gene as an internal control that is presumed to remain relatively constant across different samples. However, ideal reference genes rarely exist, and finding suitable ones is not a trivial task. It has been demonstrated that a proper choice of reference genes is highly dependent on the tissues or cells under investigation [4]. Further, reference genes are highly specific for a particular experimental model, and validation for each situation, on an individual basis, is a crucial requirement [5].

Preterm birth (delivery before 37 weeks of gestation) is a major cause of neonatal morbidity and mortality [6]. Less than half of pregnant women presenting with preterm labor end in spontaneous preterm birth (sPTB), while the remaining end in term birth (TB) on or after 37 weeks. To better understand sPTB, using gene expression microarrays, panels of RNA transcripts were systematically identified to be aberrantly expressed in the placentas [7] and maternal blood cell samples [8] collected from pregnancies undergoing sPTB. Since these preterm birth-associated transcripts are detectable in maternal whole blood, in this study, we aimed to identify reference genes suitable for normalizing RNA transcripts in the whole blood of women undergoing preterm labor.

Reference genes for normalizing RNA transcripts in the human circulatory system have been reported on patients with tuberculosis [5], schizophrenia and bipolar disorder [9], and a cohort of healthy male and female adults [10]. Nevertheless, there is a lack of similar data on pregnant women. Based on a meta-analysis of publicly available gene expression microarray datasets on 1053 blood samples, Cheng and colleagues have identified a panel of candidate reference genes for normalizing RT-qPCR data from peripheral blood across healthy, non-pregnant individuals and patients with cancer or another abnormality [11]. Yet, whether those reference genes are suitable for the normalization of data from pregnant women is unknown.

To address this paucity of relevant study, we embarked on a search for reference genes suitable for the normalization of RT-qPCR data on whole blood collected from women during their presentation of preterm labor. Similar searches for reference genes often rely on a candidate gene approach or gene expression microarray data, which is sometimes referred to as transcriptome data. Such microarray-based transcriptome studies measure RNA levels based on oligonucleotide probes, which requires prior knowledge of the RNA transcript sequences, and thus are limited essentially to the more characterized genes of known sequences. To maximize the chance of finding suitable reference genes, in this study, we expanded this search to the whole-transcriptome dataset generated by RNA-sequencing (RNA-seq), which is not limited by probes of well-characterized gene sequences. We hypothesized that the whole-transcriptome of maternal blood harbors many RNA transcripts that are expressed at relatively constant levels. Based on the RNA-seq dataset, 395 genes, comprising 458 exons, were systematically identified by detecting at a low variation across all tested maternal blood samples. Subsequently, the expression stability of the selected genes was assessed using RT-qPCR in another set of maternal blood samples. Our data suggest that the whole-transcriptome harbors gene candidates of higher expression stability than the commonly used reference genes.

In this study, RNA-seq data were interpreted at the finer resolution of exons, which are sub-regions within an expressed gene transcript. Different exons of the same gene were detected at considerably different levels of variation. Based on the exon-level RNA-seq data, one can readily pinpoint the exon with the least variation in the design of RT-qPCR assays for the quantification of candidate reference genes. Moreover, we explored how the list of 458 stably detected exons could be used as a starting point for the systematic identification of reference genes in other blood compartments and different groups of patients. Lastly, we discuss the pros and cons of our exon-level RNA-seq approach, and how it may be further improved for identifying reference genes.

## 2. Results

### 2.1. Whole-Transcriptome Profiling of Maternal Blood Samples

#### 2.1.1. Overall Results of the RNA-seq Experiment

We obtained the informed consent of, and collected peripheral whole blood samples from, pregnant women during their presentation of preterm labor. Twenty blood samples were subjected to strand-specific RNA-seq (ssRNA-seq). Forty libraries (two technical replicates per blood sample) were constructed for strand-specific pair-end cDNA sequencing on the HiSeq 4000 sequencer (Illumina, San Diego, CA, USA). Low-quality sequences and adapter sequences were trimmed by Trimmomatic, v.0.33 [12], and aligned to the reference human genome (GRCh38, GENCODE release 23 primary assembly) by the sequence alignment software Spliced Transcripts Alignment to a Reference, STAR (v2.4.2, Oxford University Press Inc., New York, NY, USA) [13]. After all filtering and mapping, two libraries with a high percentage of poor or unaligned reads were rejected and removed from further analysis. Overall, the mean number of raw reads was 159 million per sample. Of the mean 93.6 million high-quality reads, 69.2% mapped to a unique location in the reference human genome. No remarkable difference was observed across the technical replicates. All high-quality mapped reads were uploaded to the Sequence Read Archive of the National Center for Biotechnology Information under the BioProject ID PRJNA387220.

#### 2.1.2. Identification of Exons That Are Stably Detected in Maternal Blood

**Figure 1:**
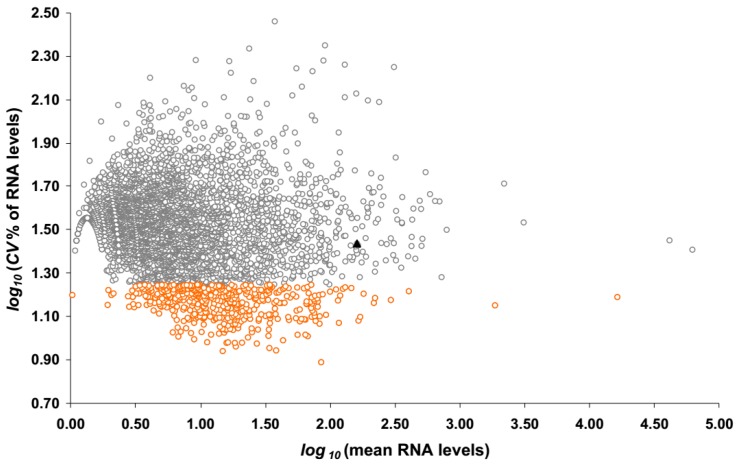
The coefficient of variation ×100% (*CV*)% and *mean* of RNA expression levels of gene exons across all maternal blood samples based on RNA-sequencing (RNA-seq). Each datapoint represents an exon. Shown are 4579 exons that are robustly detected (minimum RNA level across all samples >0 count). RNA levels are calculated by normalized read counts mapping to each exon. Highlighted in the orange circles are 458 exons with a *CV*% in the lowest 10th percentile. The black triangle shows the data from the *GAPDH* transcript.

Each gene comprises one or more sub-regions called exons. At the above sequencing depth, RNA-seq allows investigators to profile RNA levels not only at gene-level but also at the higher resolution of exon-level. Since the higher resolution exon-level data are advantageous for developing RT-qPCR assays, the read counts mapped to each exon in GRCh38 were summarized. To account for technical variations, including the differing sequencing depth and the amount of RNA input for each library, read counts were normalized using the Remove Unwanted Variation from RNA-seq data (RUVSeq) method (v1.6.0, University of California, Berkeley, CA, USA), which is based on the factor analysis of control samples, such as technical replicates [14]. Unless otherwise stated, all further instances of “counts” in this paper refer to normalized counts. Of the 11,215 exons observed, 4579 exons have at least one count in all libraries, and are considered robustly detected. For each exon, its RNA expression levels were calculated by the normalized counts of reads that mapped to that particular exonic sequence. Its mean RNA levels and coefficient of variation (*CV* = *standard deviation* divided by the *mean*) across all maternal blood samples were calculated. Figure 1 shows the *mean* and *CV* of the robustly detected exons in the *log*_10_ scale.

Without presuming a normal distribution of the linear-scale data on RNA levels across the 4579 exons, we describe the data as follows: the *median* (*interquartile range*, *IQR*) of the mean RNA levels was 5.7 counts (3.2–13 counts), and the *median* of the *CV* (*IQR*) of the RNA levels was 32.5% (24.0% to 42.0%). The *10th* and *90th percentiles* of the *CV* are 17.7% and 53.8%, respectively. The Spearman rank order correlation coefficient was only −0.06 (*p*-value, 2 × 10^−4^) between the *mean* and the *CV* of the RNA levels of these exons. Exons with a low *CV* are good candidates for reference genes, because they exhibit a low variation across all maternal blood samples (Figure 1). For example, those 458 exons (representing 395 genes) detected at a *CV* below the *10th percentile* (*log_10_*(*CV*%) < 1.25) are viable candidates (orange circles in Figure 1; File S1). The range of the *CV* of these 458 exons is 7.75–17.7%, which is considerably small compared with the *CV* of the least variably detected *GAPDH* exon (27.3%; *log*(*CV*%) = 1.4; Figure 1, black triangle). They were detected at various RNA levels ranging from 1 count to over 16,000 counts. Genes represented by these exons were considered stably detected in maternal blood.

#### 2.1.3. Pathways and Functional Annotation Terms Associated with the Genes That Are Stably Detected in Maternal Blood

**Figure 2:**
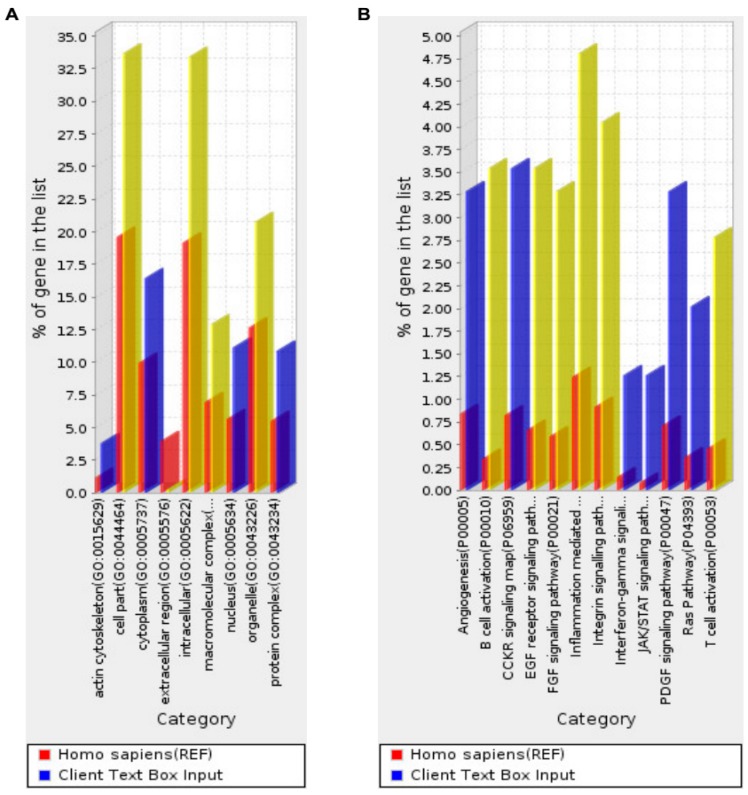
Over-represented annotation terms in the list of 395 genes that are most stably detected in maternal blood. (**A**) Over-represented protein analysis through evolutionary relationships (PANTHER) gene ontology (GO)-Slim terms in cellular components. (**B**) Over-represented PANTHER pathways. The list of genes represented by exons detected across all samples at a *CV* in the lowest *10th percentile* (orange circles in Figure 1) is subjected to the over-representation test. The percentage of genes in each category of cellular component GO-terms or pathways among this list of most stably detected genes in maternal blood (client input, blue or yellow bars) is shown. It is compared with the percentage of genes in the corresponding category among the entire list of genes in the human reference genome (REF, red bars). *p*-Values are adjusted for multiple testing by the Bonferroni method. Blue bars, *p* < 0.05. Yellow bars, *p* < 0.001. Red bar, baseline for comparison.

To gain insights into the functions of the 395 genes with exons that are stably detected in maternal blood, the enrichment of the representation of pathways or gene ontology (GO) terms [15] was statistically tested using the Protein ANalysis THrough Evolutionary Relationships (PANTHER) classification system [16,17,18]. Briefly, the percentage of genes associated with each category of pathway or GO terms among the list of stably detected genes was calculated. Then, it was compared with the percentage of genes associated with the corresponding category among the entire list of genes in the reference human genome. The fold of over-representation was reported with a *p*-value after correction for multiple testing by the Bonferroni method. Nine cellular component PANTHER GO-Slim terms were over-represented among our list of stably detected genes, as compared with the reference list of all genes in the human genome (Figure 2A). The 12 over-represented PANTHER pathways [19] are also shown (Figure 2B). The results of these and six other analyses, including the complete number of GO terms in the biological process, molecular function and cellular compartments, and the Reactome pathways [20,21], are detailed in File S2.

Compared with the reference list of all genes in the human genome, the list of 395 genes that are stably detected in maternal blood is over-represented with GO terms in cellular components (Figure 2A; File S2, PANTHER GO-Slim Cellular Component) including macromolecular complex (over-represented by 1.9-fold, adjusted *p*, 9.7 × 10^−4^), intracellular (1.7-fold; *p*, 9.2 × 10^−10^), cell part (1.7-fold; *p*, 2.3 × 10^−^^9^), organelle (1.6-fold; *p*, 2.7 × 10^−^^4^), and actin cytoskeleton (3.3-fold; *p*, 4.1 × 10^−3^). Overall, this is in line with the major components expected of blood cells after lysis in the RNA extraction step.

Moreover, this list of 395 stably detected genes is over-represented with 12 pathways (Figure 2B; File S2, PANTHER pathways) including B cell activation (10-fold; *p*, 3.0 × 10^−^^8^), T cell activation (6.1-fold; *p*, 4.7 × 10^−^^4^), inflammation mediated by a chemokine and cytokine signaling pathway (3.9-fold; *p*, 1.4 × 10^−^^4^), fetal growth factor (FGF) signaling pathway (5.6-fold; *p*, 1.6 × 10^−^^4^), epidermal growth factor (EGF) receptor signaling pathway (5.4-fold; *p*, 9.7 × 10^−5^), and integrin signaling pathway (4.4-fold; *p*, 1.8 × 10^−^^4^). The B cell activation pathway, T cell activation pathway, and inflammation mediated by a chemokine and cytokine signaling pathway highlight the important roles played by immune regulation in pregnancy [22], and the FGF signaling, EGF receptor signaling, and integrin signaling pathways mediate growth and proliferation [23,24,25]. The stable expression or detection of these genes in maternal blood is consistent with pregnancy and a growing fetus.

Furthermore, the list of stably detected genes in maternal blood is over-represented with pathways in other curated databases (File S2, Reactome pathways), including the regulation of actin dynamics for phagocytic cup formation (6.5-fold; *p*, 2.9 × 10^−3^), Fc γ receptor (FCGR)-dependent phagocytosis (6.1-fold; *p*, 7.0 × 10^−4^), and clathrin-mediated endocytosis (4.8-fold; *p*, 1.9 × 10^−2^). These are consistent with the presence of white blood cells.

#### 2.1.4. Shortlisting of Reference Genes from the RNA-Sequencing Data and the Literature

Ideally, the RNA levels of the reference gene and our gene of interest should not differ by more than a few orders of magnitude. A panel of 36 preterm birth-associated transcripts are reported to be aberrantly expressed in the preterm placenta and released into maternal plasma [7]. Of these, 12 placental transcripts were observed at a median of 5 counts in our RNA-seq data on maternal whole blood (Table S1). In addition, preterm birth-associated transcripts aberrantly expressed in maternal blood cells of the women presented with preterm labor were discovered. The RNA levels of these blood transcripts in whole blood are expected to be higher than those of the placental transcripts. The reference genes expressed at RNA levels of about 5 counts to 500 counts, and had a low *CV* across the maternal blood samples that were shortlisted. In our RNA-seq data, the *CV* of the RNA levels expressed by the *DDX17* (Ensembl exon ID, ENSE00001942031), *EXOC8* (ENSE00001442235), and *PPP1R15B* (ENSE00001443770) genes are 7.7%, 9.0%, and 9.4%, respectively. See Table 1 for the names and symbols of the genes mentioned in this paper. Their *CV* ranked 1st, 4th, and 6th among the 4579 robustly detected exons in maternal blood. Their mean RNA levels (± standard deviation (SD)) are 85 counts ± 7 counts, 34 counts ± 3 counts, and 27 counts ± 3 counts, respectively.

Additionally, other reference genes were shortlisted for normalizing the RT-qPCR data in human blood sample. Previously, other investigators also applied high-throughput data to systematically search for reference genes for normalizing RNA transcripts levels in blood. In a study based on microarray data, among several other genes, *RPL37A* was found to be suitable for normalizing RT-qPCR data in whole blood from tuberculosis patients [5]. *RPL37A* was also robustly detected. In a meta-analysis of microarray data on 1053 blood samples from healthy individuals and patients with cancer and other abnormalities, less than a dozen genes were found to be expressed at less than a *CV* of 30% [11]. Of these, *ACTB*, *HUWE1*, and *TPT1* showed the highest RNA levels. Historically, *GAPDH* was often used as a reference gene, despite the controversy over whether its expression is stable enough to serve this purpose [26].

**Figure 3:**
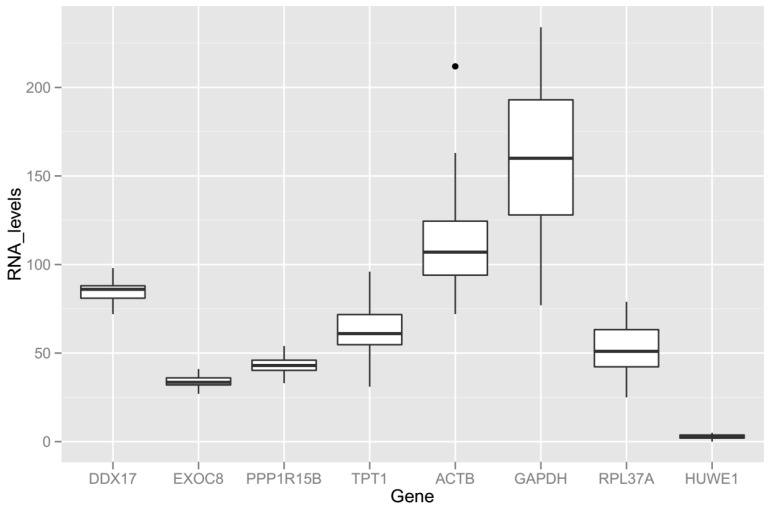
Box plot of the RNA levels of eight candidate reference genes in all maternal blood samples subjected to an RNA-seq analysis. RNA levels are calculated by normalized read counts, which have taken into account the varying RNA input, sequencing depths, and other technical variations. The bold line inside each box is drawn to the *median*, the bottom and top of each box to the *25th* and the *75th percentiles*, and the whiskers to the *10th* and *90th percentiles*. An outlier is shown as a black dot.

To investigate whether the mentioned genes are suitable for normalizing RT-qPCR data from maternal blood samples, the RNA level distribution was examined (Figure 3). As expected, the *IQR* of the RNA levels of *DDX17*, *EXOC8*, and *PPP1R15B* are small, because they were shortlisted based on their small *CV* in the same dataset. The *IQR* of the RNA levels of *GAPDH* is the largest among the shortlisted genes. The *CV* of the RNA levels for *TPT1* (ENSE00001618156), *ACTB* (ENSE00001902558), *GAPDH* (ENSE00001817977), *RPL37A* (ENSE00001900648), and *HUWE1* (ENSE00000978660) are 21%, 25%, 27%, 29%, and 43%, respectively. The highly variable expression levels of the *RPL37A* and *HUWE1* genes, and the low RNA levels expressed by the *HUWE1* gene, make them unsuitable to serve as reference genes in maternal blood samples. In fact, of these six candidates identified from the literature, the *CV* of five genes are larger than the lower quartile of the *CV* (24%), as shown by the viable reference gene candidates that may be identified from our RNA-seq dataset (Figure 1, orange circles). Nevertheless, it appears that these candidates from the literature may complement the range of RNA levels not covered by the three candidates shortlisted based on our RNA-seq data. Hence, we embarked on designing RT-qPCR assays to quantify the RNA levels of the *ACTB*, *DDX17*, *EXOC8*, *GAPDH*, *PPP1R15B*, and *TPT1* genes in maternal blood samples.

#### 2.1.5. Exon-Level RNA-Sequencing Data in Selected Genes

**Figure 4:**
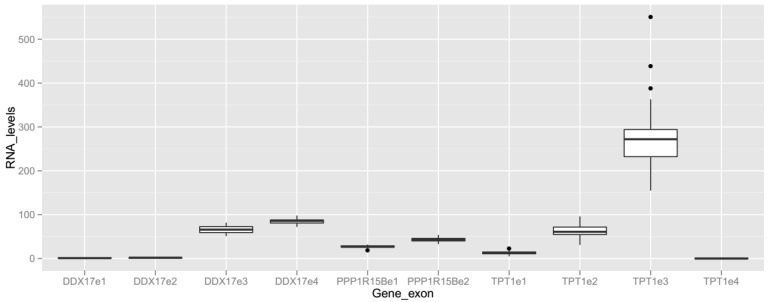
Box plot of RNA levels of multiple exons in selected genes across all blood samples subjected to an RNA-seq analysis. Selected exons (e1, e2, etc.) detected in the RNA-seq dataset are shown. See Table 2 for their Ensembl exon ID, *mean*, *standard deviation* (*SD*), and *CV* of the RNA levels. See legend of Figure 3 for the calculation of RNA levels, definition of the box, whiskers, and dots.

To observe the data distribution of RNA levels of different exons on the same gene, genes with multiple exons detected (Figure 4 and Table 2) were selected. Much variation is observed in the detection of exons from the same gene in maternal blood, with non-overlapping *IQR* (Figure 4) between them. This observation highlights the advantage of summarizing the RNA-seq data at the exon-level. In designing RT-qPCR assays to quantify the reference gene, it is advisable to target the exon detected at a lower *CV*.

The *CV* is large for exons detected at low mean RNA levels in this RNA experiment (Table 2); for example, *DDX17*e1, *DDX17*e2, and *TPT1*e4. Sequencing deeper than 150 million reads per sample may result in a more accurate counting of reads and may lower the *CV* of these exons. However, our data suggest that their expression levels are relatively lower than other exons of the same gene. RT-qPCR assays targeting these exons may be less reliable.

### 2.2. Gene Expression Stability Analysis

#### 2.2.1. Comparing Complementary Algorithms in Analyzing Gene Expression Stability

To validate whether the candidate reference genes are stably detected, gene expression was assessed by a different technology platform, namely RT-qPCR, on an independent cohort comprising 32 maternal blood samples which were not used in the RNA-seq experiment. To ensure the best experimental results, the RT-qPCR assays were designed, optimized, and performed in compliance with the Minimum Information for Publication of Quantitative Real-time PCR Experiment (MIQE) guidelines [27]. The experimental details, including the sequences of the thermal profile, the primer, and the hydrolysis probe, are described in the Methods section and File S3.

We used two common softwares, namely geNorm [4] and NormFinder [28], to analyze the expression stability of the six selected genes. In the geNorm software ((qbase+ Version 3.1, Biogazelle, Gent, Belgium), for each candidate reference gene, a stability value (*M*), which is defined as the average pairwise variation of that particular gene compared with all other candidate genes, is calculated. In the NormFinder software (v0.953, Molecular Diagnostic Laboratory, Department of Medicine, Department of Molecular Medicine, Aarhus University Hospital, Aarhus, Denmark), a stability value is also calculated for each gene by a model-based approach, taking into account its intra- and inter-group variations. In both softwares, the lowest stability value indicates the most stable gene.

**Figure 5:**
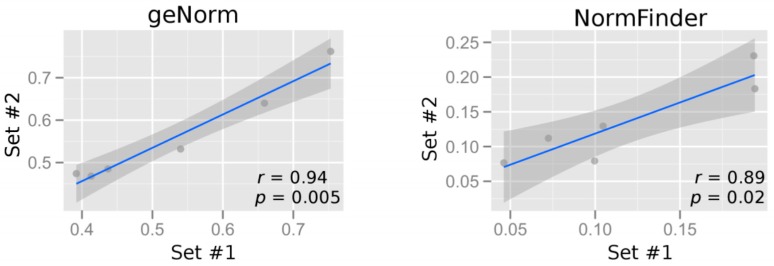
Correlation plots of gene expression stability values from the respective software in sample set #1 vs. set #2. The geNorm *M* stability value (**left**) and the NormFinder stability values (**right**) are plotted. Genes with the smallest stability value have the most stable expression. Each dot represents a gene. The entire set of samples are divided into two subsets, sets #1 and #2, each comprising approximately equal portion of sPTB cases and TB controls. Spearman correlation coefficient *r* (*ρ*) and *p* values are shown. The grey zone is the 95% confidence interval of the best-fitting line (blue).

The usual practice is to assess the stability values in a first batch of samples, and then presume that these values are applicable in other future batches of samples. To test how far this presumption is valid for maternal blood samples collected from women presenting with preterm labor, the 32 samples were partitioned into two subsets, each containing an approximately equal ratio of sPTB cases to TB controls (Table 3). Then, gene stability values were calculated by both softwares for the two subsets (Figure 5). The stability values calculated using geNorm in subsets #1 and #2 are correlated (Spearman correlation coefficient, *r* = 0.94, *p* = 0.005), and so are those calculated using NormFinder (Spearman, *r* = 0.89, *p* = 0.02). The 95% confidence interval of the best-fitting line between the two subsets in geNorm appears to be narrower than that in NormFinder (Figure 4, grey zone).

Further, the candidate reference genes were ranked in increasing order of their stability values. In other words, the most stable gene ranks first. The ranks for each gene in the two subsets and the absolute rank difference between them were tabulated (Table 4). The sum of the rank differences for all six tested genes is smaller for the stability values calculated by the geNorm software than those by the NormFinder software. Taken altogether, for the maternal blood samples and the tested candidate reference genes, geNorm gave slightly more reproducible results between the two subsets compared with NormFinder.

#### 2.2.2. Assessing Gene Expression Stability in Maternal Blood Samples

**Figure 6:**
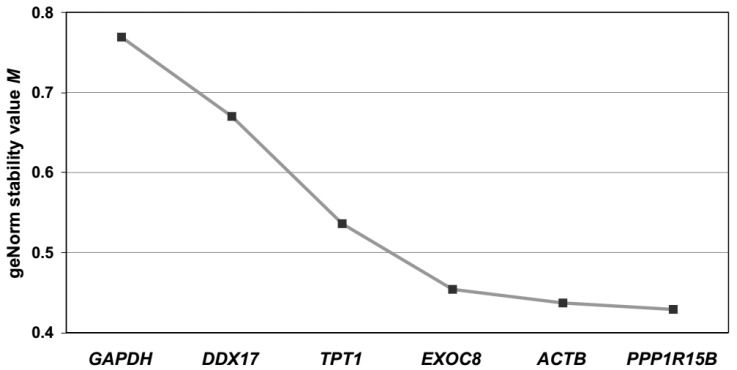
Average expression stability values (*M*) in the entire set of maternal blood samples. *M* is calculated using geNorm and the remaining control genes during stepwise exclusion of the least stable control gene. The gene with the lowest *M* has the most stable expression.

The geNorm software, which gave slightly more reproducible results, was used to analyze the entire set of 32 maternal blood samples. Based on stepwise exclusion of the least stable reference gene using the geNorm software, the stability values of the remaining control genes were plotted and the genes were ranked (Figure 6). The candidates in descending order of gene expression stability are *PPP1R15B*, *ACTB*, *EXOC8*, *TPT1*, *DDX17*, and *GAPDH*.

**Figure 7:**
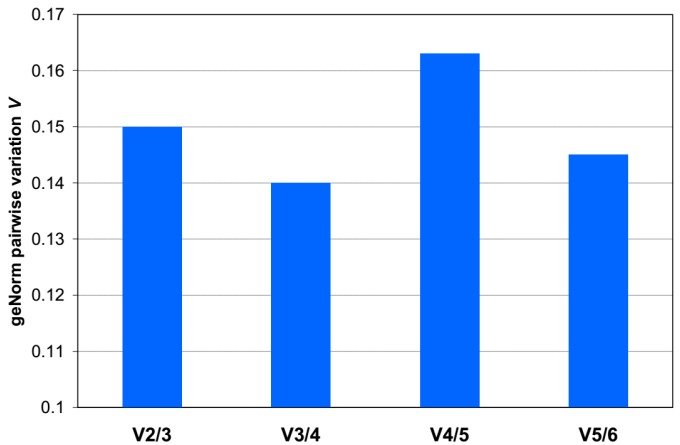
Pairwise variation analysis between normalization factors in the entire set of maternal blood samples. To determine the optimal number of reference genes required for accurate normalization, a pairwise variation (*V_n/n+_*_1_) analysis was performed between the normalization factors *NF_n_* and *NF_n+_*_1_, where *n* is the number of reference genes.

The normalization factor (*NF_n_*) of a certain number (*n*) of genes was calculated, starting with the most stable genes. To determine the possible need or utility of including a certain number of genes for normalization, the pairwise variation *V_n_*_/*n*+1_ was calculated between *NF_n_* and *NF_n_*_+1_ (Figure 7). Based on the data in the geNorm paper, if *V_n_*_/*n*+1_ > 0.15, the added gene has a significant effect and should preferably be included for the calculation of a reliable *NF* [4]. In the gene stability analysis, the *V*_2/3_ was 0.15. Thus, adding the third most stable reference gene has little effect on the *NF*_3_ compared with *NF*_2_. Hence, the optimal number of reference genes is 2. As such, the optimal *NF* can be calculated as the geometric mean of *PPP1R15B* and *ACTB*.

The data from this expression stability analysis have shown that the RNA transcripts of *PPP1R15B*, *ACTB*, *EXOC8*, and *TPT1* are expressed at relatively consistent levels in the maternal blood samples obtained from pregnant women ending in sPTB or TB.

## 3. Discussion

RNA-seq has facilitated us to search for candidate reference genes from the whole-transcriptome. Compared with a gene expression microarray, RNA-seq is not limited by a fixed number of probes and known gene sequences. Consequently, considerably more reference gene candidates were expressed and identified in this RNA-seq study (Figure 1) than in a similar microarray study [11]. However, because the amount of RNA that could be extracted from this preciously collected cohort is limited, it is impractical to systematically validate all candidates using RT-qPCR. Thus, we have validated candidates which are expressed at similar levels as our genes of interest.

RNA-seq, which was performed at a reasonable sequencing depth, has enabled us to profile the transcriptome at the higher resolution of the exon-level. As illustrated by the three selected genes in Figure 4, the exons of the same gene are detected at considerably different levels. This implies that exon-level data may guide the design of RT-qPCR assays to more specifically target a sub-region that is advantageous for the quantification of a gene transcript. In the case of designing RT-qPCR assays for reference genes, the exons with the lowest *CV* are the preferred targets.

We have also been able to assess the variations of candidate reference genes in terms of *CV* and *IQR* of the normalized read counts on the RNA-seq data. Still, there are limitations of such an assessment. The total RNA used for constructing the sequencing library contains a considerable portion of ribosomal RNA (rRNA). Its presence may affect the accuracy of the quantification of the amount of RNA input for library construction. Moreover, since the predominance of rRNA often masks the signals from other more informative mRNA in RNA-seq experiments, a common practice is to reduce the rRNA before library construction. However, such a practice contributes to a source of technical variations. Combining the quality-filtering, mapping, and normalization steps in the data analysis, each RNA-seq dataset contains a considerable amount of noise, which should be vigorously controlled for reliable gene expression profiling. Therefore, it is important to validate the findings from RNA-seq by an independent technology platform, such as RT-qPCR and cohort. This is especially true for the identification of reference genes, because the impact of technical variation in RNA-seq on a gene expression stability analysis has not been extensively studied. Furthermore, we have supplemented our RNA-seq-based selection of candidates with relevant studies which have employed another platform for profiling transcriptome.

We have used two complementary approaches for validating the expression stability of the selected reference genes. Both geNorm and NormFinder gave highly correlated (Spearman, *r* = 0.94 and 0.89, respectively) stability values between two different subsets of samples. Thus, the data suggest that both softwares generate similar stability values across different batches of samples. Although geNorm has generated slightly more correlated stability values across the two subsets, it is noted that both geNorm and NormFinder have consistently ranked *PPP1R15B*, *EXOC8*, and *ACTB* as the most stably detected genes (Table 4). Similarly, in the analysis of the entire set of 32 samples, geNorm has also ranked the same three genes as the most stably detected genes (Figure 6). Apparently, for whole blood samples collected from women during the presentation of preterm labor, *PPP1R15B*, *EXOC8*, and *ACTB* are suitable to serve as reference genes for normalizing the RNA levels of other circulating RNA transcripts. On a related note, among the genes selected for an expression stability analysis, *GAPDH* was essentially consistently ranked as the least stable by both softwares in both subsets (Table 4) and the entire set (Figure 6). Our data suggest that reference genes with lower variation than *GAPDH* do exist in maternal blood.

We observed that the RNA-seq data is not always predictive of the gene expression stability analysis data based on RT-qPCR. For instance, in the RNA-seq data, the RNA levels of *ACTB* were detected at a *CV* of 25%, which is more variable than those of *DDX17* (7.7%), *EXOC8* (9.0%), and *PPP1R15B* (9.4%). On the other hand, in the geNorm analysis, *ACTB* turned out to be one of the most stably detected genes. A closer examination revealed that the *ACTB* exons targeted by the commercially available RT-qPCR assay were not detected in our RNA-seq dataset. The RT-qPCR assay pre-designed by that company targeted an amplicon that is mapped to more than one genomic location. This type of exonic sequencing reads been filtered out after mapping, because they may interfere with the counting of transcripts in the RNA-seq experiment. This illustrates how a search for reference genes based on RNA-seq may benefit from relevant studies using an alternative technology.

The major advantage of our RNA-seq approach is that reference gene candidates could be systematically identified from the whole-transcriptome, which is more comprehensive. Moreover, the exon-level data allow a more specific design of RT-qPCR assays. The downside is that certain short sequencing reads that are mapped to more than one unique location in the genome are missed. For instance, the *ACTB* exon targeted by the commercial RT-qPCR assay was not detected in our RNA-seq data. It has been estimated that the RNA levels of hundreds of genes in the human genome could not be quantified accurately by RNA-Seq [29]. These genes are enriched for gene families, and many of them have been implicated in human disease. Usually, these ambiguously mapped reads are discarded to improve the accuracy of the quantification of the uniquely mapped reads in an RNA-seq analysis. Recently, methods for including these non-unique reads in an RNA-seq analysis have emerged. For instance, it is now possible to quantify the RNA levels of a family of similar sequences as a group [29]. Combined with the increasingly longer read length facilitated by newer sequencing platforms and reagents, we expect that this negative impact of ambiguous reads on an RNA-seq analysis will be minimized.

In this study, we provided a list of 458 exons (395 genes) that are most stably detected in maternal blood. Unlike many other tissues, peripheral blood is readily and non-invasively obtainable from a patient. Many RNA transcripts expressed in other tissues are often released into the blood circulation, including preterm birth-associated placental RNA and pregnancy-associated microRNA [7,30]. Nevertheless, the potential use of these circulating RNA as biomarkers is hindered by the paucity of study on reference genes in peripheral blood. Shortlisting from the transcriptome data and validating them by RT-qPCR are two essential but resource-demanding steps in finding suitable reference genes. Although the reference genes suitable for normalizing blood expression data from patients presenting with preterm labor may not be suitable for data from patients suffering from other conditions, our list of stably detected exons (Figure 1) could still be useful for the normalization of other experimental systems.

To find reference genes in blood samples from patients with other diseases, one may first shortlist about 10 stably detected exons from our RNA-seq data (File S1) with similar RNA levels to the gene of interest. Then, RT-qPCR assays targeting the specific exons could be designed for a gene expression stability analysis by geNorm, NormFinder, or similar software. Thus, the time and resources to perform the whole-transcriptome experiment and bioinformatics analysis can be saved. Since blood cells are the predominant source of nucleic acids in cell-free plasma [31], we reason that our RNA-seq data on whole blood will also serve as a starting point for finding reference genes in plasma.

In addition, we noted that the stably detected genes in maternal blood are over-represented with annotation terms in macromolecular complex and actin cytoskeleton (Figure 2A). This is not surprising, because they are the major structural components of any cell. Interestingly, other terms are associated with phagocytosis (File S2, Reactome pathways), B cell activation, T cell activation, and inflammation (Figure 2B). This is consistent with the notions that pregnancy and preterm birth are associated with immune regulation and infection, respectively.

Intriguingly, terms in the FGF and EGF receptor signaling pathways and Integrin pathways are also over-represented in the stably detected genes. Members of the FGF family function in the earliest stages of embryonic development and during organogenesis to maintain progenitor cells and mediate their growth, differentiation, survival, and patterning [23]. The EGF receptor signaling pathway is one of the most important pathways that regulates growth, survival, proliferation, and differentiation in mammalian cells [24]. Integrins contribute to cell growth by providing a physical linkage between cytoskeletal structures and the extracellular matrix, and also by participating in various signal transduction processes [25]. The stable detection of genes in these pathways and fetal growth warrants further investigation. Will these genes remain stably detected in the maternal blood of pregnancy complicated by intrauterine growth restriction, macrosomia, or gestational diabetes?

Analogous to the systematic search for reference genes to normalize RNA data based on RNA-seq, investigators have also searched for reference genes to normalize protein data based on proteomics. It would be interesting to contemplate whether the genes with stable RNA expression in blood, as identified in this study, may also have stable protein expression. However, in a recent study on breast cancer, a global analysis of the correlation of mRNA-to-protein yielded a median Pearson value of *r* = 0.39, with 6135 out of 9302 mRNA–protein pairs (66.0%) correlating significantly at a false discovery rate <0.05 [32]. Hence, extrapolating from mRNA expression data to protein should be interpreted with caution. Nevertheless, the current results presented are useful to serve as a resource on reference genes in the transcriptome.

Taken altogether, the maternal circulation harbors RNA transcripts that are stably detected. We identified a list of 395 genes that were detected at a low *CV* in maternal whole blood samples collected from women presenting with preterm labor. They are good reference gene candidates for normalizing expression data from these patients. Particularly, as further suggested by the results of the expression stability analysis, the RNA transcripts of *PPP1R15B*, *ACTB*, *EXOC8*, and *TPT1* are stably expressed in the maternal whole blood samples. Hence, these four genes are useful to serve as reference genes for normalizing the concentrations of other circulating transcripts in maternal peripheral whole blood.

Our list may also be useful as a starting point for finding reference genes for normalizing data from patients with other disease, from non-pregnant females or from pregnant women without preterm labor, or from samples of different blood compartments. A handful of candidates could first be shortlisted from the RNA-seq identified list of stably detected genes (File S1), particularly *PPP1R15B*, *ACTB*, *EXOC8*, and *TPT1*. Then, an expression stability analysis on the shortlisted candidates could be performed directly without going through the time- and resource-consuming screening experiments like RNA-seq or expression microarray. Since this list is based on data from human blood, we reason that the candidates proposed in this study are more suitable for normalizing RNA data in blood when compared with other lists based on tissues besides blood.

With reference genes that are more stably detected in blood, it is hoped that the hurdles of normalization could be overcome, and that more differentially expressed transcripts could be identified in the circulatory system.

## 4. Materials and Methods

### 4.1. Recruitment of Study Subjects

This study was approved by the Joint Chinese University of Hong Kong and New Territories Easter Cluster Clinical Research Ethics Committee (project CRE-2012.032, approved on 19 Jul 2013) and the Institutional Review Board of Hallym University Kangnam Sacred Heart Hospital (project 2013.10-85, approved on 31 October 2013). Women presenting with preterm labor, and that fulfilled the inclusion and exclusion criteria, were invited to participate in this study. The inclusion criteria were women with (i) uterine contractions at least once every 10 min <34 weeks, (ii) intact membrane, (iii) singleton pregnancies, and (iv) a Chinese or Korean ethnicity. The exclusion criteria were women with pregnancies complicated with (i) preterm prelabor rupture of membrane, (ii) multiple gestation, (iii) preeclampsia, (iv) fetal growth restriction, (v) macrosomia, (vi) fetal distress, (vii) antepartum hemorrhage, (viii) fetal chromosomal or structural abnormalities, (ix) history of uterine abnormality or cervical surgery, and (x) indicated preterm births before 37 weeks (induction of labor, elective or emergency term cesarean deliveries), where deliveries are iatrogenic. Gestational ages were established based on menstrual date confirmed by sonographic examination <20 weeks. Delivery outcome was traced and categorized accordingly into spontaneous preterm birth (sPTB, before 37 weeks of gestation) and term birth (TB, on or after 37 weeks) groups. Cases with chorioamnionitis were included in the sPTB group.

### 4.2. Blood Collection and RNA Extraction

Blood sample (9 mL) was collected from the antecubital fossa in ethylenediaminetetraacetic acid (EDTA)-containing tubes (Greiner Bio-One, Frickenhausen, Germany). To minimize RNA degradation, the blood sample was mixed with RNAlater (Thermo Fisher Scientific, Waltham, MA, USA) shortly after venesection and stored at −80 °C until extraction. RNA was extracted using the RiboPure RNA Purification Kit (blood) (Thermo Fisher Scientific), and treated with *DNase* I (Thermo Fisher Scientific) to minimize contaminating genomic DNA. The quality of the RNA preparation was assessed by spectrophotometry and RNA Pico chip on the Bioanalyzer (Agilent, Palo Alto, CA, USA).

### 4.3. RNA-Sequencing

Forty libraries (two technical replicates per blood sample) were constructed for strand-specific pair-end cDNA sequencing on the HiSeq 4000 sequencing platform (Illumina) (TruSeq 3000 4000 SBS Kit v3) according to the TruSeq Stranded Total RNA Sample Prep Guide (Part # 15031048 Rev. E). To minimize the highly abundant but uninformative ribosomal RNA and globin mRNA transcripts from masking the signals from the more informative transcripts, RNA samples were subjected to pre-treatment by Ribo-Zero Globin (Illumina). The remaining ribosomal RNA reads were removed (RSeQC v2.6.2) [33]. Low-quality sequences and adapter sequences were trimmed by Trimmomatic, v.0.33 [12]. The trimmed sequence reads were aligned to the reference human genome (GRCh38, GENCODE release 23 primary assembly; aligner, STAR (v2.4.2) [13]). After sequence trimming and mapping, two libraries with a high percentage of poor or unaligned reads were rejected and removed from further analysis. The counts of the aligned reads were summarized by HTSeq (v0.6.1, California Institute of Technology, Pasadena, CA, USA) [34]. To remove technical variations, the read counts were normalized using the R-package RUVSeq (ver. 1.6.0, University of California, Berkeley, CA, USA) according to instructions in the manual compiled on 3 May 2016 [14]. The RUVs method was used to estimate the unwanted variation using replicate samples. The mean and *CV* of RNA levels were calculated at the exon-level. When multiple exons were robustly detected in the RNA-seq dataset, the data from the exon with the lowest *CV* were reported unless otherwise stated. After all of the quality checking and filtering procedures mentioned were performed, RNA-seq data from a total of 19 blood samples, collected from 10 women ending in sPTB and 9 women ending in TB, were analyzed.

### 4.4. RT-qPCR

To leverage on the exon-level transcriptome data, we designed RT-qPCR assays to target the exon with the lowest *CV* of RNA levels for all except two genes in this study using the on-line PrimerQuest software (https://eu.idtdna.com/Primerquest/Home/Index) by Integrated DNA Technologies (IDT, Coralville, IA, USA). The assays for the *ACTB* and *GAPDH* genes were predesigned by and purchased from Integrated DNA Technologies, Inc. To improve the accuracy of the quantification, hydrolysis probes were used. The primers and probes were validated in silico for possible secondary structures and non-specific binding by Primer-BLAST [35]. The full probe and primer sequences, reaction conditions, and PCR efficiencies are shown in File S3. To avoid contaminating genomic DNA, the extracted RNA samples were treated with *DNase* I before RT-qPCR. To monitor for environmental contamination, no template controls were run in parallel.

A RT-qPCR primed by random primers is linear over a narrower range than a similar reaction primed by target-specific primers [36]. Further, it has been shown that the use of random hexameric primer sequences for reverse transcription may overstate the actual amount of mRNA up to 19 times [37]. Therefore, for more accurate results, gene-specific primers were used for the reverse transcription step in RT-qPCR. To minimize technical variation and the risk of contamination, one-step RT-qPCR was performed using the RNA Master Hydrolysis Probe kit (Roche, Basel, Switzerland) on the LC480 platform (Roche), which involves no opening of the reaction vessel after the addition of the RNA template. For the gene expression stability analysis, 10 ng of *DNase* I-treated RNA was added to each reaction. The geNorm analysis was carried out in qBase PLUS, which is MIQE-compliant [38]. The PCR efficiency of each assay was determined by a calibration curve constructed from standards of known concentrations. In total, RT-qPCR was performed on 32 blood samples, collected from 17 women ending in sPTB and 15 women ending in TB (Table 3).

### 4.5. Other Data Analyses

Unless mentioned above, all data analysis, including correlation tests, descriptive statistics, and graph plotting, were performed using DeduceR [39], which is a graphical user interface for R and Microsoft Excel.

## 5. Conclusions

The RNA transcripts of 395 genes in File S1, including those of *PPP1R15B*, *ACTB*, *EXOC8* and *TPT1* could be used as reference genes for normalizing the concentrations of other circulating transcripts in maternal peripheral whole blood.

## Figures and Tables

**Table 1:** Gene symbols and names mentioned in this paper.

Gene Symbol	HGNC ^1^ Approved Name	HGNC ID ^2^	Location	Accession Number
*ACTB*	*Actin β*	132	7p22.1	NM_001101
*DDX17*	*DEAD-box helicase 17*	2740	22q13.1	NM_001098504
*EXOC8*	*Exocyst complex component 8*	24,659	1q42.2	NM_175876
*GAPDH*	*Glyceraldehyde-3-phosphate dehydrogenase*	4141	12p13.31	NM_002046, NM_001289745, NM_001289746
*PPP1R15B*	*Protein phosphatase 1 regulatory subunit 15B*	14,951	1q32.1	NM_032833
*RPL37A*	*Ribosomal protein L37a*	10,348	2q35	NM_000998
*TPT1*	*Tumor protein, translationally-controlled 1*	12,022	13q14.13	NM_001286272, NM_003295, NM_001286273
*HUWE1*	*HECT, UBA and WWE domain containing 1, E3 ubiquitin protein ligase*	30,892	Xp11.22	NM_031407

^1^ HGNC, HUGO Gene Nomenclature Committee; HUGO, Human genome organization; ^2^ Identification number.

**Table 2:** Details of multiple exons in selected ^1^ genes detected in the RNA-seq dataset.

Gene Exon	Exon ID (Ensembl, GRCh38.p10)	*Mean* RNA Levels (Counts)	*SD* (Counts)	*CV*
*DDX17*e1	ENSE00001855680	1.3	0.52	40%
*DDX17*e2	ENSE00001863979	1.6	0.55	34%
*DDX17*e3	ENSE00001924900	66	9.4	14%
*DDX17*e4	ENSE00001942031	85	6.6	8%
*PPP1R15B*e1	ENSE00001443770	27	2.6	9%
*PPP1R15B*e2	ENSE00001443771	43	4.2	10%
*TPT1*e1	ENSE00001479630	13	3.6	28%
*TPT1*e2	ENSE00001618156	63	13	21%
*TPT1*e3	ENSE00001820001	272	74	27%
*TPT1*e4	ENSE00001824735	0.45	0.69	153%

^1^ See Figure 4 and text for further information.

**Table 3:** Samples used for gene expression stability analysis.^1^

	Patients Ending in sPTB ^1^ (*n*)	Patients Ending in TB ^2^ (*n*)	Total (*n*)
Subset #1	9	8	17
Subset #2	8	7	15
Entire set	17	15	32

^1^ sPTB, spontaneous preterm birth before 37 weeks. ^2^ TB, term birth on or after 37 weeks.

**Table 4:** Ranking of stability values calculated by the respective software in the two sets of samples.^1^

Gene	geNorm Stability Value			NormFinder Stability Value		
	Rank in Set #1	Rank in Set #2	Absolute Rank Difference	Rank in Set #1	Rank in Set #2	Absolute Rank Difference
*ACTB*	2	1	1	3	2	1
*DDX17*	5	5	0	6	5	1
*EXOC8*	3	3	0	1	1	0
*GAPDH*	6	6	0	5	6	1
*PPP1R15B*	1	2	1	2	3	1
*TPT1*	4	4	0	4	4	0
Total	-	-	2	-	-	4

^1^ The entire set of samples are divided into two subsets, sets #1 and #2, each comprising an approximately equal portion of sPTB cases and TB controls.

## Supplementary Materials

Supplementary materials can be found at www.mdpi.com/1422-0067/18/8/1709/s1.

## Abbreviations

ssRNA-seq	Strand-specific RNA sequencing
RT	Reverse transcription
qPCR	Quantitative polymerse chain reaction
sPTB	Spontaneous preterm birth
TB	Term birth
*CV*	*Coefficient of variation*
